# Biopolymer-Based Films Reinforced with Green Synthesized Zinc Oxide Nanoparticles

**DOI:** 10.3390/polym14235202

**Published:** 2022-11-29

**Authors:** Johar Amin Ahmed Abdullah, Mercedes Jiménez-Rosado, Antonio Guerrero, Alberto Romero

**Affiliations:** 1 Departamento de Ingeniería Química, Escuela Politécnica Superior, Universidad de Sevilla, 41011 Sevilla, Spain; 2 Departamento de Ingeniería Química, Facultad de Química, Universidad de Sevilla, 41012 Sevilla, Spain

**Keywords:** cellulose acetate, chitosan, films, gelatin, zinc oxide nanoparticles

## Abstract

Nowadays, biopolymer-based films are being developed as an alternative to conventional plastic-based films, mainly because they are non-toxic, flexible, inexpensive, and widely available. However, they are restricted in their applications due to several deficiencies in their properties. Accordingly, the reinforcement of these materials with nanoparticles/nanofillers could overcome some of their shortcomings, especially those processed by green methods. Green synthesized zinc oxide nanoparticles (ZnO-NPs) are highly suggested to overcome these deficiencies. Therefore, the main aim of this work was to develop different biopolymer-based films from cellulose acetate (CA), chitosan (CH), and gelatin (GE) reinforced with ZnO-NPs prepared by casting, and to assess their different properties. The results show the improvements produced by the incorporation of ZnO-NPs (1% *w*/*w*) into the CA, CH, and GE systems. Thus, the water contact angles (WCAs) increased by about 12, 13, and 14%, while the water vapor permeability (WVP) decreased by about 14, 6, and 29%, the water solubility (WS) decreased by about 23, 6, and 5%, and the transparency (T) increased by about 19, 31, and 20% in the CA, CH, and GE systems, respectively. Furthermore, the mechanical properties were enhanced by increasing the ultimate tensile strength (UTS) (by about 39, 13, and 26%, respectively) and Young’s modulus (*E*) (by about 70, 34, and 63%, respectively), thereby decreasing the elongation at the break (ε_max_) (by about 56, 23, and 49%, respectively) and the toughness (by about 50, 4, and 30%, respectively). Lastly, the antioxidant properties were enhanced by 34, 49, and 39%, respectively.

## 1. Introduction

A thin film (usually <1 mm thick, transparent, stretchable, and made of polyethylene or polypropylene) provides an excellent transparency, flexibility, adaptability, and impermeability, and it is perfect for packaging a variety of products of different shapes and sizes. Considering that films effectively prevent microbe interaction and oxidation–reduction reactions, these properties are of potential interest to the food industry [1,2,3,4]. A significant increase in the need for films has occurred over the last few years, as a result of safety concerns during the transportation of packages, as well as for more attractive aesthetic and hygienic products [5]. However, biodegradability problems make these packaging products unsustainable. In this way, biopolymers such as polysaccharides, polyesters, lipids, and proteins are designed to meet industrial and consumer needs, as well as to be environmentally friendly [6,7,8]. Thus, a variety of natural or natural derivative polymer-based films have been developed, such as cellulose acetate (CA), chitosan (CH), and gelatin (GE), which have a suitable biodegradability without being hazardous, offering a wide range of potential applications [9,10]. 

CA is an esterified polyester produced from a variety of cellulosic raw materials (e.g., cotton, sugarcane, rice straw, recycled paper, wood, bagasse, etc.) with several beneficial properties, including a low melting temperature, excellent optical clarity, chemical resistance, and durability [11,12]. Nevertheless, it has some limitations due to its stiffness, poor dimension stability at high temperatures, and plasticizer requirements for industrial processing [11,12]. CH is a linear cationic polysaccharide containing (β-(1 → 4)-2-amino-2-deoxy-D-glucopyranose) at a random distribution, which is derived from deacetylated units of chitin (*N*-acetyl-D-glucosamine linked via β-(1 → 4)-glycosidic linkages). As the second most important source of natural polymers after cellulose, chitin is easy to process, non-toxic, biocompatible, biodegradable, genocompatible, hemocompatible, antibacterial, and environmentally friendly. Additionally, it contains three functional groups (primary and secondary hydroxyl groups (-OH) and amine groups (-NH_2_)), making it extremely reactive in chemical reactions [13,14,15]. Nevertheless, a further investigation is necessary to maintain and enhance these properties [13]. Likewise, GE is an edible polymer protein obtained from irreversibly hydrolyzed collagen combined with functional amino acids (proline, glycine, and hydroxyproline). GE has multiple key advantages, including an easy formation, high flexibility, adequate gas barrier property, affordability, and high availability and reliability to produce efficient packaging materials [3,16,17,18]. Nonetheless, it exhibits a low mechanical and thermal resistance, high water solubility (WS), and high vapor permeability (WPV) [19,20].

Several industrial applications rely on these polymers, including their use in the cosmetic and hygienic industry for the production of hair gel shampoos and beauty products [21,22,23,24]. Furthermore, these materials offer many potential pharmaceutical and biomedical applications, including anticancer, antibiotic, antimicrobial, antidiabetic, antihypertensive, and antioxidant agents, gene therapy, tissue engineering, and wound care and healing [25,26]. They are also capable of encapsulating oils and drugs, as well as stabilizing emulsions [27,28]. Other applications include paints, glass frames, and membranes for water and fertilizer treatment [22]. However, the most important use is in food protection from several factors, including light, oxidation, and drying, by incorporating antioxidants, antimicrobials, antifungals, and nutrients to prolong their shelf life and maintain their safety [29]. Food products have a significantly reduced shelf life due to oxidation, resulting in the loss of their natural values (e.g., fatty acids, proteins, and soluble vitamins), energy content reduction, generation of undesirable flavors and odors, and pigment degradation in addition to changes in the color; all these factors contribute to a decrease in the consumer’s appeal for the food. There is a direct link between rancidity and the mentioned changes, which is caused by an auto-oxidative process involving free radical chain mechanisms. Consequently, food packaging producers are continuously looking for ways to lower the oxidation of lipids within food packaging [30].

Therefore, the potential for eco-friendly food packaging has increased the interest in nanocomposites made from biopolymer-based films in recent years. Although they are superior to conventional materials, they lack the adequate barriers and mechanical properties to resist water, water vapor, and oxygen [11]. Numerous investigations are still being conducted to highlight the properties of biopolymer-based films, one of which is the incorporation of nanomaterials as reinforcement materials [31]. In this way, several biopolymer-based films have been reinforced by several metal oxide nanoparticles (MO-NPs) [11,32,33,34,35,36,37]. Further benefits of incorporating nanofillers into polymer-based films include replacing some chemicals, thus reducing their toxicity and cost [38].

Regarding nanoparticles, it is well known that zinc oxide nanoparticles (ZnO-NPs) are among the most versatile nanoparticles used in science and medicine [39]. This is due to their highly desirable physicochemical, electronic, electrical, and electrochemical properties, as well as to their high photochemical stability [40]. In addition, ZnO-NPs have also been widely used in cosmetics and drug delivery. Various skin diseases can be treated with these nanoparticles due to their potential for UV radiation absorption in sunscreens, antimicrobials, and antibiotics, as well as many other medical products [41,42]. The U.S. Food and Drug Administration has also determined that ZnO-NPs are non-toxic and suitable for use in a wide variety of medical–industrial applications [43]. A further advantage of these nanoparticles is that they are toxic to bacteria, cancer cells, leukemia cells, and, making them highly desirable as gene-delivery biosensors, drug-delivery agents and cancer treatment agents [44]. Nevertheless, the different chemical and physical characteristics of ZnO-NPs depend mainly on the method used in their synthesis [45]. These methods include precipitation, the sedimentation processes, two-step thermal-mechanical synthesis, hydrothermal, sol-gel, electrochemical deposits, laser excision molecules, chemical steam deposition, thermal decomposition, ultrasound, microwave-assisted combustion, aluminum oxide coating, combustion, and electrical deposition [46]. Even so, these methods are costly and rely on the use of toxic chemicals and protective agents that can be hazardous to the environment [47,48,49]. Recently, researchers have been focusing on eco-friendly, simple, cost-effective, renewable, non-toxic, fast, and reproducible methods to produce metal oxide nanoparticles of different shapes and sizes with a high level of crystallinity [50,51]. These eco-friendly methods are mainly achieved with plant or fruit extracts containing high polyphenol concentrations (-OH). Polyphenolic compounds reduce metallic salts into nanoparticles of a high purity, crystallinity, and stability due to their unique properties (chemical reduction capacity, nucleophilic nature, hydrogen bonding ability, acidity, chelating abilities, polarizability, etc.) [52,53]. These properties have led to the production of nanoparticles with a suitable biocompatibility to be vastly employed in food and biomedical applications [54,55,56,57]. Nevertheless, none of the previously mentioned studies has dealt with the efficiency of the green synthesis of zinc nanoparticles using *Phoenix dactylifera* L., which is a crucial parameter to achieve significantly positive environmental and economic impacts. This includes the in-depth characterization of zinc oxide nanoparticles in terms of their particle size, shape, crystallinity, and functionality to be used in different applications, including food packaging films.

Thus, the main objective of this study was to present a viable alternative for synthesizing green zinc oxide nanoparticles and incorporating them into polymer-based films to study their effect on the properties of three different polymer-based systems. To this end, the different polymer-based films (cellulose acetate-based (CA), chitosan-based (CH), and gelatin-based (GE)) were reinforced with 1.0% of green synthesized ZnO-NPs (*w*/*w* ZnO-NPs/initial polymer weight) as an additive nanofiller, to improve some of their properties. The films were fabricated by casting and were characterized for their physicochemical (hydrophobicity, water solubility, water vapor permeability, and transparency), mechanical (ultimate tensile strength (UTS), elongation at break (ε_max_), toughness, and Young modulus (*E*)), microstructural (thickness and morphology), and functional properties (antioxidant activity).

## 2. Materials and Methods

### 2.1. Materials

Cellulose acetate (CA, 39.8% acetylation, Mv = 30,000 g × mol^−1^, DS = 2.45) and chitosan (CH, 98% deacetylation, Mv = 1.61 × 105 g × mol^−1^) were purchased from Sigma Aldrich Chemie GmbH (Riedstrasse 2, Steinheim, Germany). The food gelatin (GE, type B 200/220 g blooms, containing < 10 ppm of SO_2_) were provided by Manuel Riesgo, S.A. (Madrid, Spain). Zinc chloride (ZnCl_2_), gallic acid (C_7_H_6_O_5_), acetone (CH_3_)_2_CO, acetic acid CH_3_COOH [0.05 M], and DPPH (2,2-diphenyl-1-picrylhydrazyl) were obtained from Sigma Aldrich Chemie GmbH (Riedstrasse 2, Steinheim, Germany). All other reagents and chemicals used in this study were of an analytical quality.

### 2.2. Nanoparticles Preparation

The green ZnO-NPs were performed as described in a preprint study [58], which was developed from previous studies [59,60,61]. In brief, 20 mL of the extract (4 mg × mL^−1^ of the crude extract powder dissolved in distilled water (DW)) was added dropwise into 20 mL of ZnCl_2_ solution [0.545 g, 0.2 M]. The resulting solution (pH ≈ 6.56) was subjected to magnetic stirring at 50 °C for 2 h. Then, the precipitate was filtered with Whatman n° 1 and washed several times with DW to remove the suspended impurities. As a pretreatment, the ZnO-NPs were heated at 100 °C for 8 h. As a final treatment, the ZnO-NPs were calcined at 500 °C for 5 h.

#### Efficiency of the Process

The efficiency of the green synthesis of the ZnO-NPs was estimated by calculating the reaction theoretical yield (TY) based on the stochiometric balance of the formation mechanism of the ZnO-NPs using Equation (1):

*n* ZnCl_2_ + 2*n* R-OH + *l*H_2_O → *n* ZnO + (*l* + *n*) H_2_O+ 2*n* R-Cl (1)

where ZnCl_2_ was determined as a limiting reagent (LR) by converting the mass of ZnCl_2_ (0.545 g) to the moles as follows:

(2) $$\mathrm{Moles}\;\mathrm{of}\;{\mathrm{ZnCl}}_{2}\;=\;\left(\frac{mass\;\left(g\right)}{molar\;mass\;\left(\frac{g}{\mathrm{mol}}\right)}\right)\;=\;\left(\frac{0.545\;\left(g\right)}{136.286\;\left(\frac{g}{\mathrm{mol}}\right)}\right)\;=\;0.003998\;\mathrm{mol}$$

Therefore,

(3) $$\mathrm{RL}\;=\;\left(\frac{0.003998\;\mathrm{mol}}{n}\right)$$

The theoretical yield (TY) of ZnCl_2_ in moles is given by Equation (4):

(4) $$\mathrm{TY}\;\mathrm{in}\;\mathrm{moles}\;=\;\mathrm{RL}\;\times \;\mathrm{the}\;\mathrm{number}\;\mathrm{of}\;\mathrm{moles}\;\mathrm{of}\;\mathrm{ZnO-NPs}\;=\;\left(\frac{0.003998\;\mathrm{mol}}{n}\right)\;\times \;n$$

TY in grams = TY in moles × molar mass of ZnO-NPs = 0.003998 × 81.38 = 0.3254 g(5)

Finally, the efficiency% is calculated by dividing the actual yield (the weight of the final product, AY (g)) by the theoretical yield.

(6) $$\mathrm{Efficiency}\;(\%)\;=\;\left(\frac{AY}{TY}\right)\;\times \;100$$

### 2.3. Film Processing Method

**Figure 1:**
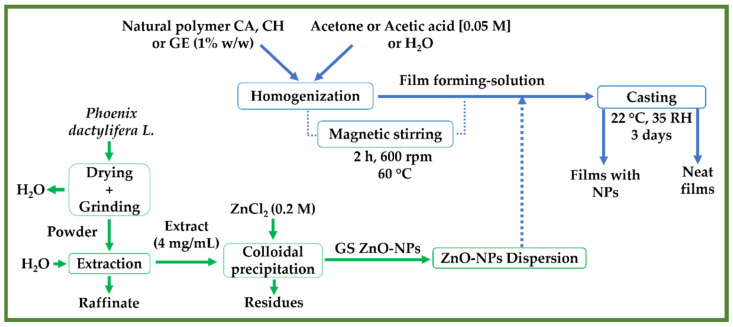
A flowchart depicting the preparation of ZnO-NPs utilizing an aqueous extract from *Phoenix dactylifera* L. and the processing of the different neat films and films reinforced with ZnO-NPs (1.0% *w*/*w*) (CA-based, CH-based, and GE-based film ZnO-NPs).

Following a previous study, the different films were fabricated by casting according to a slightly modified method [60]. Briefly, 1% *w*/*v* of the natural polymers (CA, CH, and GE) were dissolved in acetone, acetic acid 0.05 M, and distilled water, respectively. Then, each film-forming solution was stirred for 2 h at 600 rpm and 60 °C. Subsequently, 1.0% *w*/*w* of the ZnO-NPs were incorporated into the solutions via magnetic stirring for 0.5 h. Finally, an equal volume of each embedded solution (≈43 mL) was cast into Teflon plates (A ≈ 12 cm^2^) and left to dry at room conditions (22 ± 2 °C and 35% RH) for 3 days. Once the films were dry, they were peeled off and stored for a further characterization. Castings without ZnO-NPs were made as a reference. Figure 1 shows the workflow of the preparation of the ZnO nanoparticles and film processing.

## 3. Characterization Technique

### 3.1. Nanoparticles Characterization

#### 3.1.1. X-ray Diffraction (XRD)

An XRD pattern was employed to assess the crystalline system of the ZnO-NPs. The ZnO-NPs’ diffractograms were measured at 2θ = 15–70° using a Brand diffractometer (Bruker model D8 advance A25 diffractometer with Cu anode, Madrid, Spain). The size and crystallinity of the obtained ZnO-NPs were determined using the Debye–Scherrer formula, as mentioned in previous studies [59,61].

#### 3.1.2. Transmission Electron Microscopy (TEM)

The ZnO-NPs were further photographed using a TEM technique (Talos S200 microscope, FEI, Hillsboro, OR, USA) at 200 kV to investigate their size and morphology. The TEM images were obtained and subsequently labelled with Image-J (free software, Version 1.53q; NIH, Bethesda, MD, USA) to estimate the size distributions and calculate the average diameter [59].

### 3.2. Physicochemical Properties

#### 3.2.1. Water Contact Angle (WCA)

The WCA was calculated to assess the wettability of the fabricated film surfaces and hydrophobicity through an Optical Tensiometer (TL100 Attension, KSV, Helsinki, Finland) operated under sessile drop conditions. The film surface (1 × 1 cm^2^) was levelled horizontally on an adaptable stage, and then a drop of 2 µL of DW was applied onto the film surface using a micro-syringe. The WCA was calculated on both sides of the drop contour for 20 s at 12 F × s^−1^ (frames per second). The frames with a right-to-left difference greater than 2° were rejected. To ensure the reproducibility of the results, each sample was tested at least five times.

#### 3.2.2. Water Vapor Permeability (WVP)

The WVP measurements of the different films were assessed according to the ASTM method (ASTM E96 standard, 2010) [62]. Before the measurements, the films were kept in a preconditioned chamber (24 ± 1 °C and 50 ± 2% RH) for 48 h. Then, the films were placed in aluminum Payne-type test cups filled with DW to 2/3 of their internal volume. After that, the cups were mounted in a dry chamber equipped with silica gel and inflated with a gently flowing stream of dry nitrogen (N_2_) to ensure virtually no relative humidity (RH = 0%). The temperature (22 ± 2 °C) and dryness in the chamber were constantly monitored using a combined sensor. Regular weight loss monitoring of the cups (0.1 mg precision) was conducted until a steady trend was established. Then, the water vapor transmission rate (WVTR, g/h·m^2^·Pa) for a given area (A, m^2^) was determined based on the equation given below:

(7) $$WVTR\;=\;\frac{\alpha }{A}$$

where α is the slope of the straight line (R^2^ > 0.999) for weight loss over time, and (A = 0.386 × $10^{-4}$ cm^2^) presents the cup’s permeation area. Following this, a calculation of the water vapor permeance (P) was performed using Equation (8):

(8) $$P\;=\;\frac{WVTR}{∆p}\;=\;\frac{WVTR}{S\left(RH_{1}-RH_{2}\right)}$$

where ∆p is the water vapor gradient between both film surfaces, which is determined based on the pressure of the water saturation vapor at the experiment temperature (S = 2646 Pa), and $\left(RH_{1}\;=\;1\right)$ and $\left(RH_{2}\;=\;0\right)$ are the relative humidity fractions at the film surfaces exposed to the water and the chamber, respectively.

Therefore, Equation (9) was applied to calculate the water vapor permeability (WVP):

(9) WVP = P · t

where t is the film thickness in m unit.

#### 3.2.3. Water Solubility (WS)

The WS of the films was tested in accordance with previous studies with slight modifications [36,60,63,64]. In brief, each film (1 × 1 cm^2^) was initially weighed (*w_i_*) after heating in an oven at 105 °C for 24 h. After that, each film was immersed in a beaker containing 25 mL of DW for 24 h. Then, the samples were removed and dried once again at 105 °C for 24 h to determine the final dry weight (*w_f_*). Lastly, the water solubility percentage (*WS%)* was calculated as follows:

(10) $$WS\left(\%\right)\;=\;\frac{w_{i}\;-\;w_{f}}{w_{i}}\;\cdot \;100$$

#### 3.2.4. Optical Properties

The transmittance and transparency of the films were obtained according to a previous study [60]. In brief, the films were cut into pieces of 1 × 2 cm^2^ to read their transmittance at 600 nm utilizing a UV-vis spectrophotometer (Model 8451A, Hewlett Packard Co., Palo Alto, CA, USA). Thereafter, the results were calculated as the transmittance percentage at 600 nm ($T_{600}$%). A further calculation of the film transparency (*T*) was performed as described in previous research [65]:

(11) $$T\;=\;\frac{-LogT_{600}}{t}$$

where $T_{600}$ is the fraction of transmitted light across the thickness of the film (t, mm). The greater transparency index affirms the increased light absorption by the film.

### 3.3. Mechanical Properties

The mechanical characteristics of the different films were determined through tensile tests according to a slightly modified standard ISO 527-3:2019 [66]. Briefly, the samples were subjected to axial forces at 10 mm/min up to the point of breakage using a universal testing machine (MTS Insight 10, Universal Testing Machine, Darmstadt, Germany) at room conditions (22 °C and 35% RH). Accordingly, the mechanical parameters, i.e., the ultimate tensile strength (the material’s capability to withstand the maximum stress when stretched or pulled without breaking, UTS or Ϭ_max_, MPa), elongation at the break (the material’s resistance to change in shape without cracking, ε_max_, mm/mm), toughness (kJ/m^3^), and Young’s modulus (*E*, MPa) were evaluated [67].

### 3.4. Morphological Properties

A scanning electron microscopy (SEM, Zeiss EVO microscope, Pleasanton, CA, USA) magnified by 3000× and accelerated at 10 kV was used to photograph the morphological and microstructural characteristics of the different film surfaces, as well as their thicknesses [68]. In addition, the film thicknesses were labelled with Image-J (free software, Version 1.53q; NIH, Bethesda, MD, USA).

### 3.5. Functional Properties

To determine whether the films were effective as antioxidants in inhibiting the formation of free radicals, the free radical DPPH test was used according to previous protocols [36,60]. Briefly, 1 mL of DPPH (methanolic solution 40 ppm) was tested against 1 mL of the film-forming solution by mixing and incubating for 0.5 h at room conditions. The absorbance of each mixture was read at 517 nm utilizing a spectrophotometer. Furthermore, to provide a standard positive control of the experiment, gallic acid was employed and the DPPH inhibition percentage *IP* (%) was obtained from Equation (12).

(12) $$IP\;\left(\%\right)\;=\;\left(\frac{a\;-\;b}{a}\right)\;\times \;100$$

where *a* describes the absorbance of the neat DPPH solution (without any film-forming solution as an antioxidant additive), and *b* describes the absorbance of the affected DPPH (with the neat film-forming solution or film-forming solution containing ZnO-NPs).

### 3.6. Statistical Analysis

Each sample was measured at least three times in this work. The results of the statistical analyses, which were evaluated using the IBM SPSS software (IBM Corp, Armonk, NY, USA. Released 2019. IBM SPSS Statistics for Windows, Version 26.0. Armonk, NY, USA, IBM Corp), are presented as the mean and standard deviation (M ± SD) for each variable. Additionally, a one-way ANOVA was performed to estimate the heterogeneity of variance and significant differences (95% statistical confidence level and *p* < 0.05).

## 4. Results

### 4.1. Nanoparticles Characterization

#### 4.1.1. Process Efficiency

The efficiency of this green synthesis is a crucial parameter that must be taken into account to achieve significantly positive environmental and economic impacts. The reported efficiency (%) for green nanoparticles has ranged between 40 and 80% [69]. According to the actual yield of this study, the green ZnO-NPs exhibited an efficiency of 67.9%. Nevertheless, the lower yield of green synthesis remains disadvantageous when compared to the conventional synthesis of the nanoparticles, which can yield more than a 90% efficiency. However, this was found to be affected by a variety of factors, including the reaction conditions and the pre- and post-treatment periods [70]. An in-depth investigation of the nanoparticles’ synthesis must consider their properties, including the nanoparticles’ size, shape, functionality, and other properties [69]. In this way, the nanoparticles were studied by XRD and TEM to determine their size and morphology, which are essential factors that affect their sustainability and potential applications.

#### 4.1.2. XRD

**Figure 2:**
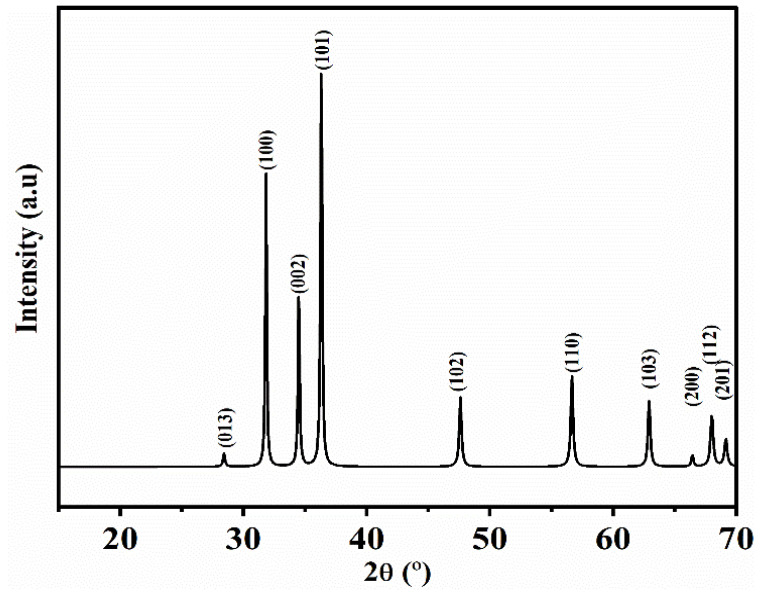
XRD spectra (standard ZnO diffraction pattern; JCPDS n°.01-079-2205) of ZnO-NPs synthesized utilizing an aqueous extract from *Phoenix dactylifera* L.

Figure 2 depicts the X-ray diffractogram of ZnO-NPs synthesized using *Phoenix dactylifera* L. extract. As can be observed, the peaks at 2θ (°) = 28.41, 31.68, 34.37, 36.29, 47.45, 56.56, 62.89, 66.43, 67.87, and 69.13° were found to be attributed to the hexagonal structures of zinc oxide, corresponding to the crystallographic reflection planes (013), (100), (002), (101), (102), (110), (103), (200), (112), and (201), respectively (standard ZnO diffraction pattern; JCPDS n°.01-079-2205) [71].

According to the Debye–Scherrer equation, the average size of the ZnO-NPs was found to be 18.6 ± 1.3 nm with ≈ 84% of crystallinity.

#### 4.1.3. TEM

**Figure 3:**
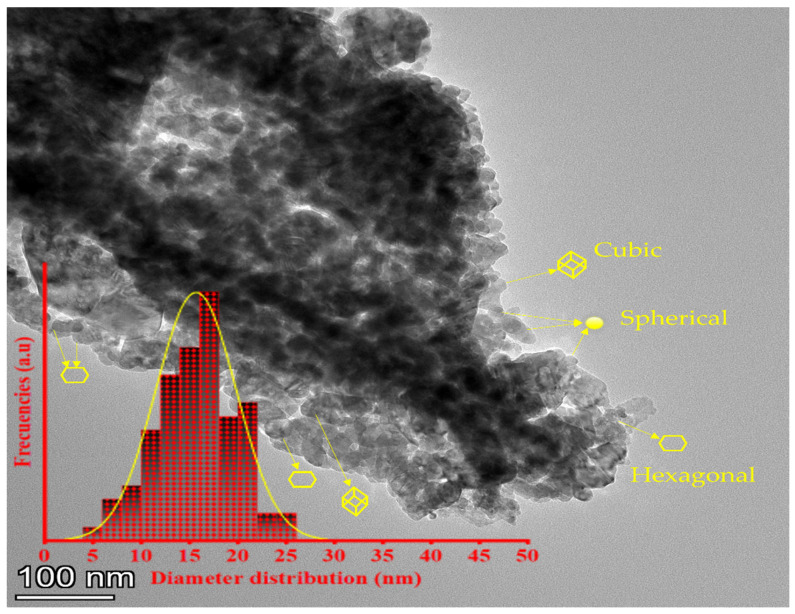
Transmission electron microscopy (TEM) of the ZnO-NPs synthesized utilizing an aqueous extract from *Phoenix dactylifera* L.

The histogram of the average diameter distributions (Lorentz curve was used to fit the histogram) and the morphology of the ZnO-NPs are depicted in Figure 3. The average diameter was found to be 16.5 ± 1.9 nm. As can be seen, the ZnO-NPs exhibited spherical, hexagonal, and cubic structures. These NPs also showed some elongated crystals with slight interactions between zinc ions on the surface of the ZnO-NPs and phenolic groups (-OH^−^), which resulted in the aggregation of the nanoparticles. Similar aggregations were observed in previous studies with green NPs [51,72].

### 4.2. Film Characterization

#### 4.2.1. Physicochemical Properties

##### Water Contact Angle (WCA)

**Figure 4:**
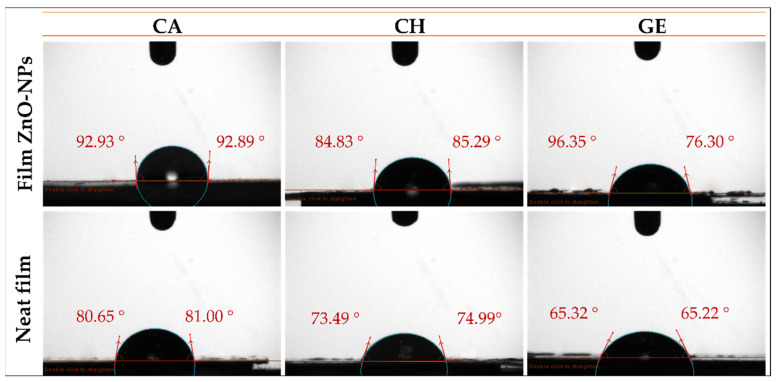
Water contact angle photographs of the different films reinforced with ZnO-NPs (1.0% *w*/*w*) (CA-based, CH-based, and GE-based film ZnO-NPs). Neat films without ZnO-NPs (CA-based, CH-based, and GE-based neat films) were added as reference.

The WCA of the food packaging films is used to determine the wettability of the film surface and evaluate its hydrophobic/hydrophilic character [65]. A hydrophilic surface is characterized by a smaller drop contact angle (an acute angle inside the drop or <90°), whereas a hydrophobic surface is characterized by a greater drop contact angle (obtuse angles or >90°) [65,73]. Figure 4 displays the WCAs of neat and composite films with ZnO-NPs, while Table 1 presents their respective mean values. Accordingly, several factors can affect the WCA values, including the polymer concentration and nature and nanoparticle content. For cellulose acetate, the CA-based neat films showed a predominant hydrophilic character, as the average WCA was ca. 80.8°. This value may confirm the effect of the polymer concentration on the hydrophilic behavior of a thin film [74,75]. In contrast, other studies have reported hydrophobic behaviors of CA-based films [76,77]. The hydrophobic behavior of CA-based neat film prepared at 2% (*w*/*v* in acetone) was also reported in a previous study with the WCA ≈ 93° [78]. However, upon the incorporation of ZnO-NPs into the CA-based film, its surface character changed from hydrophilic to hydrophobic, since the WCA increased to 92.1, indicating the hydrophobicity of ZnO-NPs. Similar results have been reported regarding the contribution of nanoparticles that lead to the enhancement of the hydrophobicity or impairment of the hydrophilicity of the membrane surfaces with increasing WCA values [73], such as the incorporation of silica nanoparticles (Si-NPs) into CA/CH-based films (CA/CH-Si) [79], and Cu-NPs into CA-based films [80]. On the other hand, the CH-based neat film seemed to be more hydrophilic, with the WCA ≈ 74.2°. This may be related to the low concentration of chitosan (1%, *w*/*v*), since the chitosan backbone is known to behave hydrophobically, especially at high concentrations [81]. When the CH-based film was embedded with ZnO-NPs, the hydrophilicity of the film was reduced to ≈85.1°. CH-based films reinforced with other nanoparticles have shown a similar behavior [82]. For the gelatin system, the GE-based neat film exhibited hydrophilic behavior, with the WCA = 65.3°, owing to the configuration of its hydrophilic groups within the framework. The hydrophilic behavior of gelatin was further explained by the orientation of the hydrophobic chains at the air-exposed surface of gelatin during the process of gelation and solvent evaporation [83]. When the ZnO-NPs were incorporated into the GE-based film, a reduction in the hydrophilicity was observed (WCA ≈ 76.3°), in consonance with the hydrophobic nature of the ZnO-NPs. In this sense, a crosslink complex may be formed by metallic oxide nanoparticles’ ions and bonded to macromolecules via multiple mechanisms, including hydrophobic interactions, hydrogen, and ionic bonds [84]. Interestingly, Roy et al. (2022) investigated the effect of ZnO-NPs at two different concentrations (1 and 2% *w*/*w* of the initial gelatin weight) on the WCA of a gelatin/cellulose nanofiber-based film (GE/CNF) containing GE (4% *w*/*v*), CNF (1% *w*/*w*), and glycerol (30% *w*/*w*). They found that the WCA of the GE/CNF-based film increased significantly from ≈ 59° to ≈ 65° with the reinforcement of 1% (*w*/*w*) of ZnO-NPs, although it did not show a significant increase after increasing the concentration to 2% (WCA ≈ 65°) [85]. This could be due to the use of contrasting concentrations (hence the high concentration of glycerol and hydrophilic GE, and the low concentration of CNF), the larger nanoparticle size, and the unknown distribution, as was mentioned above. These results are also in line with other results reported in previous studies [83,86].

##### Water Vapor Permeability (WVP)

The WVP of packaging materials is an issue of growing concern in the food packaging industry. The ZnO-NPs were assessed for their effect on the permeability of composite films by measuring their water vapor transmission rate (WVTR). A summary of the results for the WVTR, P, and WVP values is provided in Table 1. A significant reduction in the WVTR, P, and WVP values of the composite films was observed when the ZnO-NPs were incorporated into the films. Thus, the WVP of the CA-based film embedded with ZnO-NPs was reduced by about 14% (≈1.4 g·m/h·m^2^·Pa × $10^{-6}$) with respect to the WVP of the CA-based neat film (Table 1). In contrast, the WVP of the CH-based film reinforced with ZnO-NPs increased by 6% as compared with the CH-based neat film (≈1.5 g·m/h·m^2^·Pa × $10^{-6}$, Table 1). This was attributed to the heterogeneous orientation and distribution of the ZnO-NPs within the film. Previous studies have shown that several factors affect the water vapor permeability of the composite films, such as the hydrophobicity and hydrophilicity of both the polymer and nanofiller, film thickness, surface roughness, compaction, nanoparticle size, degree of crystallinity, distribution, and orientation of the nanoparticles [7,83,87]. On the other hand, the WVP of the GE-based film embedded with ZnO-NPs was reduced by about 30% (≈0.7 g·m/h·m^2^·Pa × $10^{-6}$) with respect to the WVP of the GE-based neat film (Table 1). This behavior results from the formation of highly interconnected 3D networks [88]. Nanoparticles dispersed in a polymeric matrix can generate twisted pathways that retain water molecules [64]. This factor, along with the restricted mobility of protein chains in the gelatin matrix, contributes to preventing water molecules from passing through the film [82,89]. It has been reported that switching the cation of the filler may have a marginal effect on the hydrophobic/hydrophilic balance of the filler. The strong interactions generated between the nanofillers and polymer chains lead to the consumption of hydrophilic compounds, thereby reducing the water transfer [90]. According to previous studies, the nanoparticles can reduce the water vapor permeability of the films either by lessening the free hydroxy compounds or by increasing the crystallinity and hydrophobicity of the film matrix, thus improving the water resistance of the film [10,91].

##### Water Solubility (WS)

WS is one of the most important aspects of food packaging. Consequently, insoluble films are necessary to ensure product safety and moisture resistance [36,64]. According to Table 1, the neat and composite films have different WS% values. Thus, the CA-based film embedded with ZnO-NPs displayed the lowest WS (reduced from 19.9 to 15.3%) due to an enhanced CA water insolubility by the hydrophobic ZnO-NPs [92]. The WS was found to decrease with the incorporation of cellulose nanofillers into the CA-based film [12]. Likewise, the WS of the CH-based film was reduced from 29.5 to 27.8% thanks to the ZnO-NPs’ incorporation. A similar finding has been obtained with the incorporation of SiO_2_-NPs-GA into CH-based films, which could also be explained by the hydrophobic nature of chitosan chains [75]. On the other hand, the GE-based neat film displayed the highest WS (WS = 93.2%), which can be attributed to the hydrophilic nature of gelatin [93]. Nevertheless, a significant reduction in this value was observed with the incorporation of ZnO-NPs (88.2%). Consequently, ZnO-NPs, due to their hydrophobic nature, improved the water resistance of the films by reducing their solubility in water. A similar decrease in the WS% (from 72 to 63%) was found with the incorporation of 6% (*w*/*w*) of CH-NPs into the GE-based film [64]. Nevertheless, some of the findings in the literature are contradictory; for example, the WS% has been reported to increase from 46 to 58% when GE/CH-based films were reinforced with cap-${\mathrm{Fe}}^{+3}$-HMOF-5 (capsaicin-${\mathrm{Fe}}^{+3}$ doped hollow metal-organic frameworks) [94]. Herein, all the films have shown a reduction in the water absorption in the presence of ZnO-NPs, possibly due to the hydrogen bonds formed between the ZnO-NPs and polymer chains [93]. Therefore, incorporating hydrophobic nanoparticles within polymer chains could increase the number of hydrophobic compounds due to the hydrogen bonds’ formation, thereby increasing the water resistance [95]. Regarding the different polymer-based neat films, the corresponding water solubility values were influenced by the hydrophilicity/hydrophobicity character, in line with the measurements of the water contact angle.

##### Optical Properties

A summary of the results for the transmittance ($T_{600}$%) and transparency (*T*) values is provided in Table 1. Accordingly, a significant decrease in the $T_{600}$% value and an increase in the *T* index were observed with the incorporation of the ZnO-NPs. Thus, the CA-based film was found to be the least transparent, since the CA-based neat film exhibited a $T_{600}$% of 27.8%. The further incorporation of ZnO-NPs reduced the $T_{600}$% to 16.9%. According to the film thicknesses, the *T* value of the CA-based neat film was significantly increased from 6.4 to 7.9 (*p* < 0.05). This is probably related to the higher content of solid material in the polymer matrix. For the chitosan system, the CH-based neat film also exhibited an intermediate $T_{600}$%, which was decreased by occupying the free spaces generated during the formation of the film. Therefore, the *T* value (opacity) of the embedded films with ZnO-NPs increased significantly from 2.7 to 3.9 (Table 1, *p* < 0.05). In addition, the $T_{600}$% of the GE-based neat film was reduced from 78.9% to 61.5% as a result of the ZnO-NPs’ reinforcement, where the *T* index increased significantly from 2.0 to 2.5 (Table 1, *p* < 0.05). This may be due to the presence of solid NPs dispersed in the matrix, which restrict the mobility of their chains. In this way, nanofillers dispersed with the polymer chains may occupy the vacant space and prevent light from passing across the film. An analysis by Siddique et al. (2018) showed similar results when different amounts of zinc oxide nanoparticles (1 mg and 10 mg) were added to co-polymer (40 gelatin:60 chitosan)-based films. Thus, the transparency value was found to increase from 1.7 to 2.0 and 2.2 with 1 and 10 mg of ZnO-NPs, respectively [96]. Numerous findings were also found in the literature with similar results [32,36,65,97,98].

#### 4.2.2. Mechanical Properties

**Figure 5:**
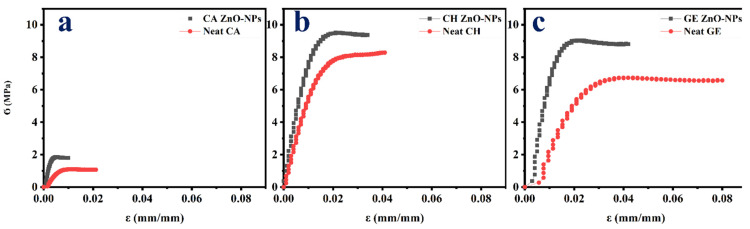
Tensile test profiles of the different films reinforced with ZnO-NPs (1.0% *w*/*w*). (**a**). CA-based, (**b**). CH-based, and (**c**). GE-based film ZnO-NPs. Neat films without ZnO-NPs (CA-based, CH-based, and GE-based neat films) were added as reference.

Figure 5 depicts the tensile profile of neat films and films reinforced with ZnO-NPs. Additionally, their mechanical parameters are summarized in Table 2. Accordingly, the neat films displayed a short elastic area followed by a longer plastic area. A significant reduction in the plastic zone was achieved when the ZnO-NPs were embedded within the films. The films embedded with ZnO-NPs exhibited an increased brittleness, due to the increase in the ultimate tensile strength (UTS, MPa) and Young’s modulus (*E*, MPa), and to the decrease in the elongation at the break (ε_max_, mm/mm) and toughness (kJ/m^3^). The UTS and *E* CA-based neat films were the lowest, which is likely due to the plasticizer requirement during the cellulose acetate processing [11,12]. A similar result was reported for the embedded cellulose containing magnetite nanoparticles [99]. Likewise, the UTS and *E* of the CH-based and GE-based neat films increased due to the ZnO-NPs’ incorporation (Table 2). The films embedded with immiscible nanoparticles may be restricted in their extensibility due to the non-homogeneous network formation within the film [100]. Therefore, the mechanical resistance of the polymer-based films embedded with NPs may be enhanced through the formation of hydrogen bonds with NPs [36]. Additionally, these results may also be due to the nanoparticle size and type, according to a previous study [60], since smaller nanoparticles lead to a greater ultimate tensile strength and Young’s modulus, owing to the enhanced interconnection in the structure and the increased strength of the network formed by nanoparticles and polymer chains [101]. Nevertheless, the ε_max_ and toughness were reduced when the films were embedded with ZnO-NPs. Consequently, the incorporation of nanoparticles stiffens the film matrix as more solid material is added [36]. Furthermore, the ZnO-NPs incorporation into the polymer films lead to a reduction in the cohesion forces between the polymer chains, resulting in a reduction in the elongation at the break [102]. Nevertheless, the incorporation of 1% ZnO-NPs resulted in optimal mechanical properties. Above this loading concentration, the nanoparticles tend to agglomerate/aggregate, leading to a decrease in the mechanical properties, as demonstrated in previous studies. For example, according to Roy et al. (2022), the increase in loading ZnO-NPs from 1 to 2% into GE/CNF-based films did not significantly increase the mechanical parameters [85]. An interesting study by Mehmood et al. (2020) incorporated iron oxide nanoparticles into GE-based films at different concentrations (5, 10, 15, and 20% *w*/*w*) and demonstrated significant improvements of up to 10%, followed by a decrease in the mechanical properties [36]. Furthermore, the amorphous networks of films and protein arrangements are hindered by nanoparticles of a hydrophobic nature that aggregate over high loading concentrations [103].

#### 4.2.3. Morphological Properties

##### Scanning Electron Microscopy (SEM)

**Figure 6:**
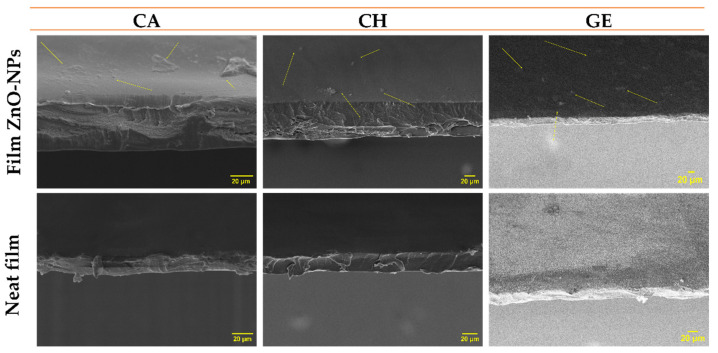
Scanning electron microscopy (SEM) images for the thickness and surfaces of the different films reinforced with ZnO-NPs (1.0% *w*/*w*) (CA-based, CH-based, and GE-based film ZnO-NPs). Neat films without ZnO-NPs (CA-based, CH-based, and GE-based neat films) were added as reference.

Roughness is an essential parameter that can facilitate or inhibit macromolecule adsorption on polymer surfaces [104]. Figure 6 illustrates the thickness and surface morphology of neat and composite films. With the inclusion of ZnO-NPs (1% *w*/*w*), the film surfaces changed from homogeneous and smooth to irregular and rougher. Several factors may contribute to this result, such as the nanoparticle sizes, granulations, dispersions, and aggregation on the film surface, which can occur during the process of solvent evaporation [65,83]. Some slight aggregation or agglomeration was observed on the surfaces of all the systems containing nanoparticles. This is due to the charge interaction between the polyphenolic groups present on the ZnO-NPs surfaces and the polymer chains of the films that may alter their structure [105,106]. Additionally, the nature of the solvent may affect the aggregation or agglomeration of the nanoparticles; therefore, the exchange rate between the solvent and non-solvent can be slowed down or accelerated [74].

#### 4.2.4. Functional Properties (Antioxidant Activity)

DPPH free radicals were used to evaluate the antioxidant properties of neat and composite films. In addition, gallic acid was used as a positive control, showing *IP%* = 95%. As can be observed in Table 2, the DPPH inhibition percentages *(IP%)* were determined. Thus, the presence of ZnO-NPs induced an increase in the antioxidant activity, possibly due to their ability to confer antioxidant properties to the environment in which they are incorporated [36,107]. Regarding the different films, an increase was obtained in the DPPH inhibition with the incorporation of ZnO-NPs to *IP% =* 57.4%, with respect to the *IP% =* 37.9% obtained for the CA-based neat film. However, some studies have indicated that cellulose derivatives have negligible antioxidant properties. For example, the DPPH inhibition was about 2% for carboxymethyl cellulose-based films, as reported by Roy et al. (2020). Therefore, they incorporated 1 wt% of curcumin and 1 wt% ZnO-NPs, obtaining *IP%* ≈ 4 and 40%, respectively [108]. Likewise, the CH-based neat film exhibited moderate antioxidant properties, increasing *IP%* from 42.8 to 83.5% in the presence of ZnO-NPs. This is due to the simultaneous activity of chitosan-free amino-functional compounds and ZnO-NPs, which trap the free radicals to produce highly stable macro-free radicals and ammonium groups [75]. Some studies have reported an inhibition of 18% by pure chitosan (2% *w*/*v* in 1% acetic acid), increasing to 92% with the incorporation of green silicon dioxide nanoparticles (SiO_2_-NPs, 8 mg/mL) [75]. Based on these results, it may be possible to determine the effect of the type and concentration of solvent and nanofiller employed in the preparation of the films [74,75]. Meanwhile, the GE-based neat film showed the lowest *IP% =* 24.9% (Table 2). Under similar conditions, this result exceeds the *IP%* of gelatin films (5% *w*/*v*; 5% of glycerol *w*/*w*) reported by Mehmood et al. (2020). Thus, the *IP%* of the gelatin films increased from ≈6% (without additives) to ≈31% (with chemical iron oxide nanoparticles, ≈7 nm, 10% *w*/*w*) [36]. In this study, the *IP%* of the gelatin reinforced by ZnO-NPs (1% *w*/*w*) was much greater (*IP% =* 40.7%). It has been suggested that higher concentrations of polyphenols in ZnO-NPs may be responsible for the increase in the DPPH inhibition observed in the presence of these nanoparticles [60]. Green synthesized nanoparticles (including low concentrations, e.g., 1%) with higher antioxidant properties can contribute to the antimicrobial properties, as already demonstrated in previous studies [60]. Given that the ZnO-NPs were produced using a green method, they demonstrated advantages in both the synthesis process and the production of the nanoparticles, increasing their utility and reducing toxic waste.

## 5. Conclusions

The zinc oxide nanoparticles prepared based on the green method using the polyphenolic extract of *Phoenix dactylifera* L. exhibited an efficiency of 67.9%, with well-dispersed and polycrystalline nanostructures (size ≈ 16–19 nm and crystalline content > 83%), which could fit a variety of purposes. ZnO-NPs are an ideal coating material for food packaging due to their several characteristics. However, the materials used in food packaging have a wide range of deficiencies, such as inadequate physicochemical, mechanical, morphological, and functional properties.

This study demonstrates that ZnO-NPs enhance the properties of cellulose, chitosan, and gelatin polymer-based films. Consequently, ZnO-NPs enhanced the hydrophobicity of the films, which in turn increased the water contact angle, reduced their water solubility, and thus reduced the water vapor permeability. A further advantage of incorporating ZnO-NPs is that they increase the thickness of the film and the solid material content, resulting in a reduction in the transmission of light, leading to an increase in the opacity. Moreover, these nanoparticles have increased the stiffness and roughness of the films. Additionally, the use of ZnO-NPs as antioxidant additives enhanced the antioxidant properties of these films. Thus, the ZnO-NPs were able to inhibit DPPH free radicals by ≈43–84%, with a concentration of 1% (*w*/*w*). Considering these benefits, the polymers embedded with ZnO-NPs offer a range of advantages as functional packaging materials with antioxidant capacities.

To the best of our knowledge, there is no study that addresses the efficiency of the green synthesis of zinc nanoparticles using *Phoenix dactylifera* L., which is a crucial parameter to achieve significantly positive environmental and economic impacts. This includes the in-depth characterization of zinc oxide nanoparticles in terms of the particle size, shape, and crystallinity to be used in different applications, including food packaging films. Thus, these factors demonstrate a significant effect on the properties of films.

However, a further investigation is necessary regarding the cytotoxicity and the possible migration of nanoparticles in healthcare and food packaging applications. Future studies will also focus on the biodegradability and thermal stability of these films, as they are expected to be environmentally friendly. Computational simulation could be taken into account in these new trials, since it offers several advantages compared to the experimental perspective, such as faster results and lower costs.

## Figures and Tables

**Table 1:** Physicochemical and optical parameters of the different films reinforced with ZnO-NPs (1.0% *w*/*w*) (CA-based, CH-based, and GE-based film ZnO-NPs). Neat films without ZnO-NPs (CA-based, CH-based, and GE-based neat films) and commercial values were added as reference. The same superscript letters (a–f) in each column indicate homogeneity of variances (*p* < 0.05).

Sample	*WCA* (°)	*WVTR* (g/h·m^2^)	P $(g/h\cdot m^{2}\cdot \mathrm{Pa})\;\times \;10^{-2}$	*WVP* $(g\cdot m/h\cdot m^{2}\cdot \mathrm{Pa})\;\times \;10^{-6}$	*WS* (%)	*T*_600_ (%)	*T*
Commercial value	96	4–23	1 × $\;10^{-2}\text{–}$1 × $10^{-4}$	1 $\times 10^{-6}\text{–}1\times 10^{-8}$	<1%	-	-
CA ZnO-NPs	92.1 ± 0.1 ^a^	80.3 ± 0.7 ^e^	3.35 ± 0.02 ^d^	1.21 ± 0.01 ^d^	15.3 ± 0.6 ^f^	16.9 ± 0.6 ^f^	7.91 ± 0.04 ^a^
Neat CA	80.8 ± 0.2 ^c^	139.9 ± 1.0 ^a^	5.25 ± 0.04 ^a^	1.41 ± 0.02 ^c^	19.9 ± 1.4 ^e^	27.8 ± 0.9 ^e^	6.43 ± 0.01 ^b^
CH ZnO-NPs	85.1 ± 0.3 ^b^	82.9 ± 0.5 ^d^	3.13 ± 0.02 ^e^	1.62 ± 0.02 ^a^	27.8 ± 0.9 ^d^	30.2 ± 0.4 ^d^	3.94 ± 0.04 ^c^
Neat CH	74.2 ± 1.1 ^e^	98.4 ± 1.8 ^c^	3.73 ± 0.07 ^c^	1.52 ± 0.05 ^b^	29.5 ± 0.2 ^c^	59.0 ± 0.7 ^c^	2.72 ± 0.02 ^d^
GE ZnO-NPs	76.3 ± 0.1 ^d^	69.9 ± 1.8 ^f^	2.61 ± 0.07 ^f^	0.71 ± 0.01 ^f^	88.2 ± 1.3 ^b^	61.5 ± 0.6 ^b^	2.51 ± 0.02 ^e^
Neat GE	65.3 ± 0.1 ^f^	111.4 ± 2.3 ^b^	4.22 ± 0.09 ^b^	1.01 ± 0.07 ^e^	93.2 ± 1.7 ^a^	78.9 ± 1.4 ^a^	2.02 ± 0.01 ^f^

**Table 2:** Results for thicknesses, mechanical parameters (UTS, ε_max_, toughness and *E*), and DPPH inhibition *IP* (%) of the different films reinforced with ZnO-NPs (1.0% *w*/*w*) (CA-based, CH-based, and GE-based film ZnO-NPs). Neat films without ZnO-NPs (CA-based, CH-based, and GE-based neat films) were added as reference. The same superscript letters (a–f) in each column indicate homogeneity of variances (*p* < 0.05).

Sample	Thickness (µm)	UTS (MPa)	ε_max_ (mm/mm)	Toughness (kJ/m^3^)	*E* (MPa)	DPPH *IP* (%)
CA ZnO-NPs	40.2 ± 1.9 ^b^	1.8 ± 0.1 ^e^	0.010 ± 0.002 ^e^	10 ^f^	180.8 ± 7.8 ^d^	57.4 ± 5.2 ^b^
Neat CA	26.3 ± 0.9 ^e^	1.1 ± 0.1 ^f^	0.022 ± 0.001 ^c^	20 ^e^	55.1 ± 1.3 ^f^	37.9 ± 0. 7 ^d^
CH ZnO-NPs	50.0 ± 3.4 ^a^	9.5 ± 0.2 ^b^	0.031 ± 0.001 ^d^	260 ^d^	316.7 ± 9.0 ^b^	83.5 ± 0.1 ^a^
Neat CH	39.3 ± 1.1 ^b^	8.3 ± 0.4 ^c^	0.040 ± 0.002 ^b^	270 ^c^	207.5 ± 1.7 ^c^	42.8 ± 1.0 ^c^
GE ZnO-NPs	28.9 ± 0.2 ^c^	9.0 ± 0.1 ^a^	0.041 ± 0.001 ^d^	300 ^b^	225.0 ± 12.5 ^a^	40.7 ± 1.4 ^c^
Neat GE	24.5 ± 1.7 ^d^	6.7 ± 0.4 ^d^	0.081 ± 0.012 ^a^	430 ^a^	83.8 ± 7.9 ^e^	24.9 ± 0.3 ^e^

## Data Availability

The data presented in this study are available on request from the corresponding author.

## Abbreviations

*E*	Young’s modulus
A	Area
AY	Actual yield
CA	Cellulose acetate
CH	Chitosan
DMSO	Dimethyl sulfoxide anhydrous
DPPH	2,2-diphenyl-1-picrylhydrazyl
DS	Degree of substitution
DW	Distilled water
Film ZnO-NPs	Film embedded with zinc oxide nanoparticles
GAE	Gallic acid equivalent
GE	Gelatin
*IP* (%)	Inhibitory concentration percentage
LR	Limiting reagent
NPs	Nanoparticles
pH	Potential or power of hydrogen
*Phoenix dactylifera* L.	Phoenix dactylifera leaves
RH	Relative humidity
T	Transparency
TY	Theoretical yield
UTS	Ultimate tensile stress
WCA	Water contact angle
WS	Water solubility
WVP	Water vapor permeability
WVTR	Water vapor transmission rate
XRD	X-ray diffraction
ZnO-NPs	Zinc oxide nanoparticles
ε_max_	Elongation at break
P	Permeance
